# *N*′-[(*E*)-Benzyl­idene]-1-ethyl-7-methyl-4-oxo-1,4-dihydro-1,8-naphthyridine-3-carbohydrazide

**DOI:** 10.1107/S1600536809048739

**Published:** 2009-11-21

**Authors:** Farah Deeba, Misbahul Ain Khan, Muhammad Zia-ur-Rehman, Ertan Şahin, Nagihan Çaylak

**Affiliations:** a Applied Chemistry Research Centre, PCSIR Laboratories Complex, Ferozpure Road, Lahore 54600, Pakistan; b Department of Chemistry, Islamia University, Bahawalpur, Pakistan; c Department of Chemistry, Faculty of Science, Atatürk University, 25240 Erzurum, Turkey; d Department of Physics, Sakarya University, Sakarya, Turkey

## Abstract

In the title compound, C_19_H_18_N_4_O_2_, the 1,8-naphthyridine ring system is essentially planar [r.m.s. deviation = 0.011 (3) Å]. The dihedral angle between the naphthyridine ring system and the phenyl ring is 28.95 (7)°. The carbohydrazide H atom is involved in an intra­molecular N—H⋯O hydrogen bond, forming a six-membered hydrogen-bonded ring. In the crystal, the mol­ecules arrange themselves into centrosymmetric dimers by means of inter­molecular C—H⋯O hydrogen bonds.

## Related literature

For the synthesis of heterocyclic compounds, see: Chen *et al.* (2001 ▶); Zia-ur-Rehman *et al.* (2006 ▶, 2009 ▶). For their biological activity, see: Ferrarini *et al.* (2000 ▶); Hoock *et al.* (1999 ▶); Nakatani *et al.* (2001 ▶); Roma *et al.* (2000 ▶). For related structures, see: Catalano *et al.* (2000 ▶); Deeba *et al.* (2009 ▶).
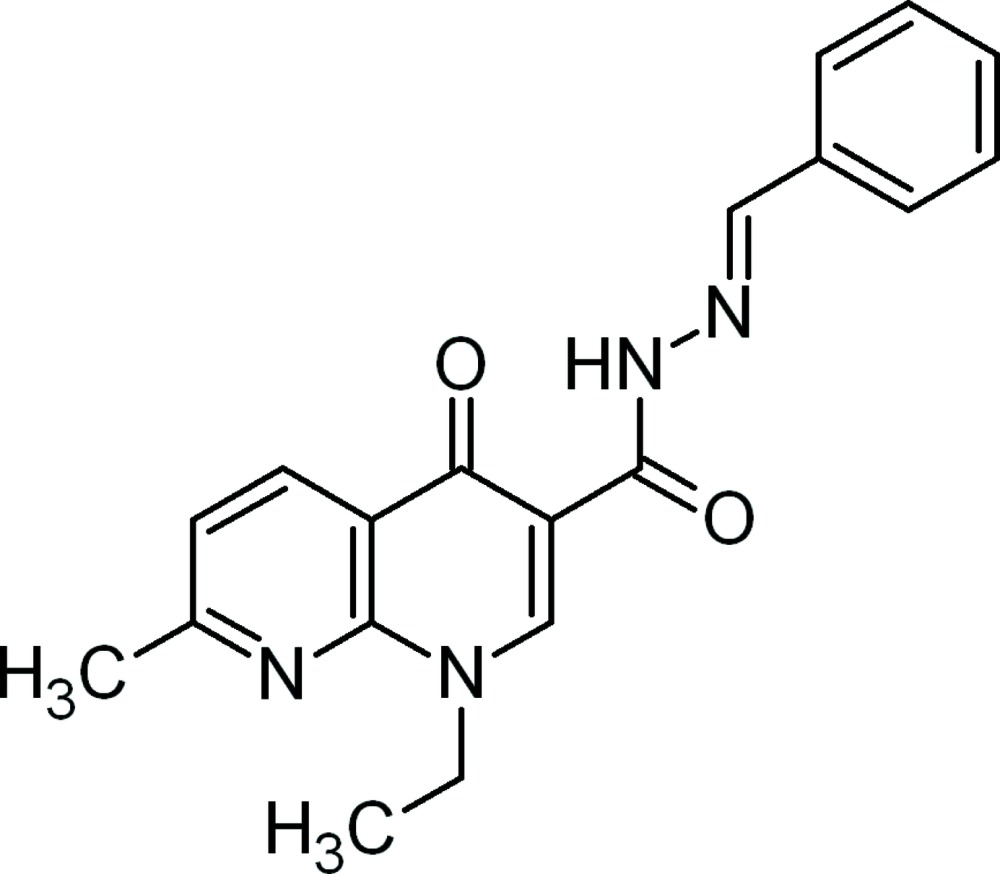


## Experimental

### 

#### Crystal data

C_19_H_18_N_4_O_2_
*M* *_r_* = 334.37
Triclinic, 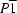

*a* = 7.1642 (1) Å
*b* = 8.8383 (1) Å
*c* = 14.4560 (2) Å
α = 82.624 (6)°
β = 85.454 (7)°
γ = 68.594 (5)°
*V* = 844.63 (4) Å^3^
*Z* = 2
Mo *K*α radiation
μ = 0.09 mm^−1^
*T* = 293 K
0.20 × 0.10 × 0.10 mm

#### Data collection

Rigaku R-AXIS RAPID-S diffractometer
Absorption correction: multi-scan (Blessing, 1995 ▶) *T*_min_ = 0.983, *T*_max_ = 0.991
18153 measured reflections
3446 independent reflections
2105 reflections with *I* > 2σ(*I*)
*R*_int_ = 0.066

#### Refinement

*R*[*F* ^2^ > 2σ(*F* ^2^)] = 0.060
*wR*(*F* ^2^) = 0.175
*S* = 1.03
3446 reflections
236 parameters
H atoms treated by a mixture of independent and constrained refinement
Δρ_max_ = 0.19 e Å^−3^
Δρ_min_ = −0.17 e Å^−3^

### 

Data collection: *CrystalClear* (Rigaku/MSC, 2005 ▶); cell refinement: *CrystalClear*; data reduction: *CrystalClear*; program(s) used to solve structure: *SHELXS97* (Sheldrick, 2008 ▶); program(s) used to refine structure: *SHELXL97* (Sheldrick, 2008 ▶); molecular graphics: *ORTEP-3 for Windows* (Farrugia, 1997 ▶); software used to prepare material for publication: *WinGX* (Farrugia, 1999 ▶) and *PLATON* (Spek, 2009 ▶).

## Supplementary Material

Crystal structure: contains datablocks I, global. DOI: 10.1107/S1600536809048739/is2485sup1.cif

Structure factors: contains datablocks I. DOI: 10.1107/S1600536809048739/is2485Isup2.hkl

Additional supplementary materials:  crystallographic information; 3D view; checkCIF report

## Figures and Tables

**Table 1:** Hydrogen-bond geometry (Å, °)

*D*—H⋯*A*	*D*—H	H⋯*A*	*D*⋯*A*	*D*—H⋯*A*
N1—H1*N*⋯O1	0.89 (3)	1.93 (2)	2.674 (3)	140 (2)
C7—H7*B*⋯O2^i^	0.97	2.45	3.204 (3)	134
C9—H9⋯O2^i^	0.93	2.51	3.340 (3)	149

Symmetry code: (i) 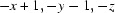.

**Fig. 1.**
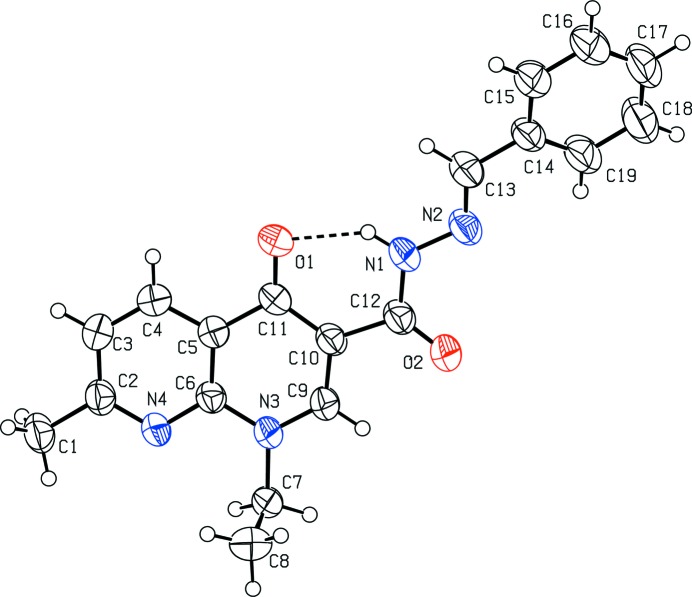
An ORTEP-3 (Farrugia, 1997) drawing of the title molecule with the atom-numbering scheme. The displacement ellipsoids are drawn at the 50% probability level.

**Fig. 2.**
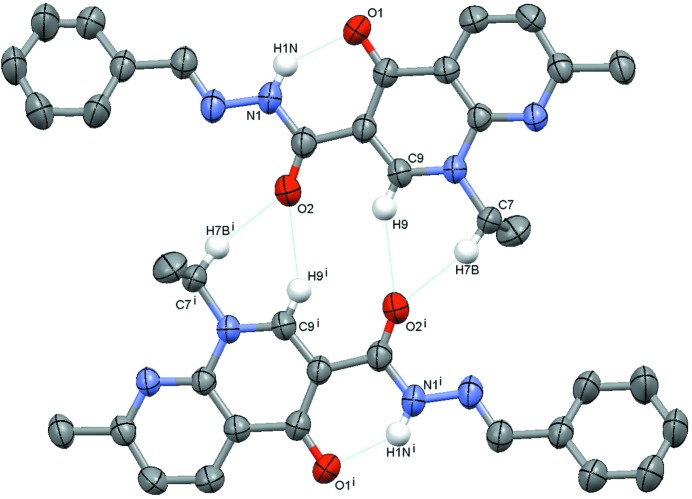
Perspective view of the three-dimensional crystal packing showing hydrogen-bonded interactions (dashed lines). [Symmetry code: (i) -x + 1, -y - 1, -z.] H atoms not involved in the hydrogen bonds have been omitted for clarity.

## supplementary crystallographic information

### Comment

1,8-Naphthyridines have been cited in the literature for their medical uses such as antibacterial (Chen *et al.*, 2001), anti-inflammatory (Roma *et al.*, 2000), anti-hypertensive and anti-platelet activities (Ferrarini *et al.*, 2000) agents. Besides few among these have been reported to be excellent fluorescent markers of nucleic acids (Hoock *et al.*, 1999) and probe molecules (Nakatani *et al.*, 2001). In continuation of our work on the synthesis, biological activity and crystal structures of various heterocyclic compounds (Zia-ur-Rehman *et al.*, 2006, 2009), we herein report the synthesis and crystal structure of the title compound (I) (Fig. 1).

The structure of the adjoined pyridine rings comprising of the naphthyridine ring is planar while carbonyl oxygen O1 on C11 is involved in intramolecular hydrogen bonding with N1H, giving rise to a six-membered hydrogen bond ring (Table 1). All bond distances are essentially identical to those found in the literature (Catalano *et al.*, 2000; Deeba *et al.*, 2009). Each molecule forms centrosymmetric dimer through intermolecular C—H···O hydrogen bonds, giving rise the formation of two six-membered hydrogen bond rings per dimer (Fig. 2).

### Experimental

A mixture of 1-ethyl-7-methyl-4-oxo-1,4-dihydro-1,8-naphthyridine-3-carbohydrazide (10.0 mmol, 2.46 g), benzaldehyde (11.0 mmol, 1.17 g), *ortho* phosphoric acid (2 drops) and ethyl alcohol (20.0 ml) was refluxed for a period of two hours. After completion of the reaction as indicated by TLC, three fourth of the solvent was evaporated and the contents were cooled to room temperature. Crystals obtained were washed with cold ethanol and dried; Yield: 89%.

### Refinement

H atoms were placed in geometrically idealized positions (C—H = 0.93–0.97 Å) and treated as riding, with *U*_iso_(H) = 1.2*U*_eq_(methine and methylene C) or 1.5*U*_eq_(methyl C). The H atoms attached to atoms N1 and C13 were located in a difference Fourier map and refined freely.

### Crystal data

**Table:**

C_19_H_18_N_4_O_2_	*Z* = 2
*M**_r_* = 334.37	*F*(000) = 352
Triclinic, *P*1	*D*_x_ = 1.315 Mg m^−^^3^
Hall symbol: -P 1	Mo *K*α radiation, λ = 0.71073 Å
*a* = 7.1642 (1) Å	Cell parameters from 18153 reflections
*b* = 8.8383 (1) Å	θ = 2.5–26.4°
*c* = 14.4560 (2) Å	µ = 0.09 mm^−^^1^
α = 82.624 (6)°	*T* = 293 K
β = 85.454 (7)°	Needles, yellow
γ = 68.594 (5)°	0.20 × 0.10 × 0.10 mm
*V* = 844.63 (4) Å^3^	

### Data collection

**Table:**

Rigaku R-AXIS RAPID-S diffractometer	3446 independent reflections
Radiation source: fine-focus sealed tube	2105 reflections with *I* > 2σ(*I*)
graphite	*R*_int_ = 0.066
ω scans	θ_max_ = 26.4°, θ_min_ = 2.5°
Absorption correction: multi-scan (Blessing, 1995)	*h* = −8→8
*T*_min_ = 0.983, *T*_max_ = 0.991	*k* = −11→11
18153 measured reflections	*l* = −18→18

### Refinement

**Table:**

Refinement on *F*^2^	Primary atom site location: structure-invariant direct methods
Least-squares matrix: full	Secondary atom site location: difference Fourier map
*R*[*F*^2^ > 2σ(*F*^2^)] = 0.060	Hydrogen site location: inferred from neighbouring sites
*wR*(*F*^2^) = 0.175	H atoms treated by a mixture of independent and constrained refinement
*S* = 1.03	*w* = 1/[σ^2^(*F*_o_^2^) + (0.0691*P*)^2^ + 0.1621*P*] where *P* = (*F*_o_^2^ + 2*F*_c_^2^)/3
3446 reflections	(Δ/σ)_max_ < 0.001
236 parameters	Δρ_max_ = 0.19 e Å^−^^3^
0 restraints	Δρ_min_ = −0.17 e Å^−^^3^

### Special details

**Table:**

Geometry. All s.u.'s (except the s.u. in the dihedral angle between two l.s. planes) are estimated using the full covariance matrix. The cell s.u.'s are taken into account individually in the estimation of s.u.'s in distances, angles and torsion angles; correlations between s.u.'s in cell parameters are only used when they are defined by crystal symmetry. An approximate (isotropic) treatment of cell s.u.'s is used for estimating s.u.'s involving l.s. planes.
Refinement. Refinement of *F*^2^ against ALL reflections. The weighted *R*-factor *wR* and goodness of fit *S* are based on *F*^2^, conventional *R*-factors *R* are based on *F*, with *F* set to zero for negative *F*^2^. The threshold expression of *F*^2^ > 2σ(*F*^2^) is used only for calculating *R*-factors(gt) *etc*. and is not relevant to the choice of reflections for refinement. *R*-factors based on *F*^2^ are statistically about twice as large as those based on *F*, and *R*- factors based on ALL data will be even larger.

### Fractional atomic coordinates and isotropic or equivalent isotropic displacement parameters (Å^2^)

**Table:**

	*x*	*y*	*z*	*U*_iso_*/*U*_eq_	
C1	−0.1188 (4)	0.2780 (3)	0.28444 (16)	0.0632 (7)	
H1C	−0.1027	0.1871	0.3312	0.095*	
H1A	−0.2588	0.3349	0.2734	0.095*	
H1B	−0.0637	0.3511	0.3056	0.095*	
C8	0.3626 (5)	−0.3046 (4)	0.26006 (19)	0.0813 (9)	
H8A	0.4932	−0.3472	0.2304	0.122*	
H8C	0.3494	−0.3818	0.3109	0.122*	
H8B	0.3459	−0.2034	0.2834	0.122*	
H13	0.603 (4)	−0.009 (3)	−0.3488 (17)	0.072 (8)*	
H1N	0.454 (4)	−0.035 (3)	−0.2036 (17)	0.065 (8)*	
C5	0.1821 (3)	0.1032 (3)	0.03208 (15)	0.0469 (5)	
O1	0.3049 (3)	0.1303 (2)	−0.12329 (11)	0.0627 (5)	
N4	0.0597 (3)	0.0569 (2)	0.19056 (12)	0.0476 (5)	
N3	0.2269 (3)	−0.1657 (2)	0.10789 (12)	0.0466 (5)	
N1	0.4915 (3)	−0.1421 (3)	−0.20600 (13)	0.0546 (5)	
C10	0.3530 (3)	−0.1374 (3)	−0.04983 (14)	0.0468 (5)	
C9	0.3224 (3)	−0.2282 (3)	0.03032 (15)	0.0477 (5)	
H9	0.3716	−0.3413	0.0308	0.057*	
N2	0.5729 (3)	−0.2165 (3)	−0.28556 (13)	0.0574 (5)	
C11	0.2838 (3)	0.0374 (3)	−0.05407 (15)	0.0480 (5)	
C6	0.1544 (3)	0.0025 (3)	0.11057 (14)	0.0444 (5)	
O2	0.5063 (3)	−0.3819 (2)	−0.12262 (11)	0.0682 (5)	
C2	−0.0111 (3)	0.2171 (3)	0.19557 (16)	0.0510 (6)	
C3	0.0108 (4)	0.3277 (3)	0.12111 (17)	0.0631 (7)	
H3	−0.0395	0.4393	0.1266	0.076*	
C13	0.6167 (4)	−0.1254 (4)	−0.35312 (17)	0.0580 (6)	
C7	0.2050 (4)	−0.2758 (3)	0.19052 (15)	0.0564 (7)	
H7B	0.2152	−0.3795	0.1709	0.068*	
H7A	0.0733	−0.2281	0.2199	0.068*	
C14	0.6940 (4)	−0.1896 (3)	−0.44275 (15)	0.0549 (6)	
C4	0.1069 (4)	0.2702 (3)	0.04011 (17)	0.0594 (7)	
H4	0.1221	0.3431	−0.0097	0.071*	
C12	0.4574 (4)	−0.2336 (3)	−0.12865 (15)	0.0522 (6)	
C15	0.7825 (4)	−0.1063 (3)	−0.50867 (17)	0.0681 (7)	
H15	0.7952	−0.0104	−0.4953	0.082*	
C19	0.6743 (5)	−0.3296 (4)	−0.46494 (18)	0.0778 (9)	
H19	0.6135	−0.3863	−0.4219	0.093*	
C17	0.8318 (5)	−0.3036 (4)	−0.6144 (2)	0.0901 (10)	
H17	0.8780	−0.3421	−0.6720	0.108*	
C16	0.8518 (5)	−0.1637 (4)	−0.59383 (19)	0.0807 (9)	
H16	0.9123	−0.1073	−0.6373	0.097*	
C18	0.7436 (6)	−0.3872 (4)	−0.5502 (2)	0.0992 (12)	
H18	0.7305	−0.4826	−0.5640	0.119*	

### Atomic displacement parameters (Å^2^)

**Table:**

	*U*^11^	*U*^22^	*U*^33^	*U*^12^	*U*^13^	*U*^23^
C1	0.0648 (16)	0.0627 (16)	0.0549 (15)	−0.0110 (13)	0.0065 (12)	−0.0216 (12)
C8	0.108 (2)	0.0676 (18)	0.0590 (16)	−0.0244 (17)	−0.0082 (16)	0.0081 (14)
C5	0.0490 (13)	0.0456 (12)	0.0439 (12)	−0.0144 (10)	0.0011 (10)	−0.0061 (10)
O1	0.0816 (13)	0.0578 (10)	0.0443 (9)	−0.0239 (9)	0.0122 (8)	−0.0018 (8)
N4	0.0495 (11)	0.0484 (11)	0.0412 (10)	−0.0126 (9)	0.0058 (8)	−0.0109 (8)
N3	0.0524 (11)	0.0450 (10)	0.0398 (10)	−0.0149 (9)	0.0093 (8)	−0.0096 (8)
N1	0.0641 (13)	0.0592 (13)	0.0389 (11)	−0.0206 (11)	0.0121 (9)	−0.0124 (9)
C10	0.0482 (13)	0.0518 (13)	0.0381 (11)	−0.0155 (10)	0.0046 (10)	−0.0082 (10)
C9	0.0492 (13)	0.0460 (12)	0.0448 (12)	−0.0133 (10)	0.0070 (10)	−0.0111 (10)
N2	0.0586 (13)	0.0661 (13)	0.0432 (11)	−0.0171 (10)	0.0096 (9)	−0.0136 (9)
C11	0.0504 (13)	0.0540 (13)	0.0392 (11)	−0.0192 (11)	0.0038 (10)	−0.0063 (10)
C6	0.0421 (12)	0.0461 (12)	0.0408 (11)	−0.0112 (10)	0.0033 (9)	−0.0070 (9)
O2	0.0869 (13)	0.0542 (11)	0.0533 (10)	−0.0148 (9)	0.0182 (9)	−0.0144 (8)
C2	0.0487 (13)	0.0503 (13)	0.0493 (13)	−0.0099 (11)	0.0003 (10)	−0.0133 (11)
C3	0.0777 (18)	0.0438 (13)	0.0589 (15)	−0.0108 (13)	0.0029 (13)	−0.0105 (11)
C13	0.0620 (16)	0.0665 (17)	0.0463 (14)	−0.0239 (13)	0.0064 (11)	−0.0119 (12)
C7	0.0746 (17)	0.0453 (13)	0.0436 (12)	−0.0187 (12)	0.0179 (12)	−0.0054 (10)
C14	0.0554 (15)	0.0638 (15)	0.0403 (12)	−0.0161 (12)	0.0055 (10)	−0.0076 (11)
C4	0.0732 (17)	0.0480 (14)	0.0499 (14)	−0.0157 (12)	0.0022 (12)	−0.0011 (11)
C12	0.0525 (14)	0.0578 (15)	0.0435 (13)	−0.0162 (12)	0.0070 (10)	−0.0111 (11)
C15	0.0797 (19)	0.0685 (17)	0.0563 (15)	−0.0290 (15)	0.0143 (13)	−0.0099 (13)
C19	0.110 (2)	0.0807 (19)	0.0517 (15)	−0.0485 (18)	0.0251 (15)	−0.0146 (14)
C17	0.125 (3)	0.095 (2)	0.0570 (17)	−0.048 (2)	0.0380 (17)	−0.0298 (16)
C16	0.095 (2)	0.089 (2)	0.0568 (16)	−0.0363 (18)	0.0282 (15)	−0.0125 (15)
C18	0.154 (3)	0.095 (2)	0.0663 (19)	−0.066 (2)	0.038 (2)	−0.0343 (17)

### Geometric parameters (Å, °)

**Table:**

C1—C2	1.501 (3)	C9—H9	0.9300
C1—H1C	0.9600	N2—C13	1.279 (3)
C1—H1A	0.9600	O2—C12	1.220 (3)
C1—H1B	0.9600	C2—C3	1.397 (3)
C8—C7	1.501 (4)	C3—C4	1.369 (3)
C8—H8A	0.9600	C3—H3	0.9300
C8—H8C	0.9600	C13—C14	1.467 (3)
C8—H8B	0.9600	C13—H13	1.01 (3)
C5—C4	1.391 (3)	C7—H7B	0.9700
C5—C6	1.398 (3)	C7—H7A	0.9700
C5—C11	1.467 (3)	C14—C19	1.375 (4)
O1—C11	1.246 (3)	C14—C15	1.384 (3)
N4—C2	1.328 (3)	C4—H4	0.9300
N4—C6	1.345 (3)	C15—C16	1.378 (3)
N3—C9	1.339 (3)	C15—H15	0.9300
N3—C6	1.388 (3)	C19—C18	1.379 (4)
N3—C7	1.477 (3)	C19—H19	0.9300
N1—C12	1.354 (3)	C17—C16	1.367 (4)
N1—N2	1.379 (3)	C17—C18	1.370 (4)
N1—H1N	0.89 (3)	C17—H17	0.9300
C10—C9	1.373 (3)	C16—H16	0.9300
C10—C11	1.435 (3)	C18—H18	0.9300
C10—C12	1.494 (3)		
			
C2—C1—H1C	109.5	C3—C2—C1	120.2 (2)
C2—C1—H1A	109.5	C4—C3—C2	119.4 (2)
H1C—C1—H1A	109.5	C4—C3—H3	120.3
C2—C1—H1B	109.5	C2—C3—H3	120.3
H1C—C1—H1B	109.5	N2—C13—C14	120.2 (2)
H1A—C1—H1B	109.5	N2—C13—H13	123.6 (14)
C7—C8—H8A	109.5	C14—C13—H13	116.3 (14)
C7—C8—H8C	109.5	N3—C7—C8	111.6 (2)
H8A—C8—H8C	109.5	N3—C7—H7B	109.3
C7—C8—H8B	109.5	C8—C7—H7B	109.3
H8A—C8—H8B	109.5	N3—C7—H7A	109.3
H8C—C8—H8B	109.5	C8—C7—H7A	109.3
C4—C5—C6	116.1 (2)	H7B—C7—H7A	108.0
C4—C5—C11	121.7 (2)	C19—C14—C15	118.3 (2)
C6—C5—C11	122.2 (2)	C19—C14—C13	121.3 (2)
C2—N4—C6	117.81 (19)	C15—C14—C13	120.3 (2)
C9—N3—C6	119.42 (18)	C3—C4—C5	120.2 (2)
C9—N3—C7	119.91 (18)	C3—C4—H4	119.9
C6—N3—C7	120.65 (17)	C5—C4—H4	119.9
C12—N1—N2	119.2 (2)	O2—C12—N1	123.7 (2)
C12—N1—H1N	117.7 (16)	O2—C12—C10	122.0 (2)
N2—N1—H1N	123.0 (16)	N1—C12—C10	114.2 (2)
C9—C10—C11	120.18 (19)	C16—C15—C14	120.8 (3)
C9—C10—C12	115.2 (2)	C16—C15—H15	119.6
C11—C10—C12	124.6 (2)	C14—C15—H15	119.6
N3—C9—C10	124.7 (2)	C14—C19—C18	120.9 (3)
N3—C9—H9	117.7	C14—C19—H19	119.5
C10—C9—H9	117.7	C18—C19—H19	119.5
C13—N2—N1	115.6 (2)	C16—C17—C18	120.0 (3)
O1—C11—C10	125.0 (2)	C16—C17—H17	120.0
O1—C11—C5	120.8 (2)	C18—C17—H17	120.0
C10—C11—C5	114.23 (19)	C17—C16—C15	120.0 (3)
N4—C6—N3	116.26 (19)	C17—C16—H16	120.0
N4—C6—C5	124.4 (2)	C15—C16—H16	120.0
N3—C6—C5	119.32 (18)	C17—C18—C19	120.0 (3)
N4—C2—C3	122.0 (2)	C17—C18—H18	120.0
N4—C2—C1	117.8 (2)	C19—C18—H18	120.0
			
C6—N3—C9—C10	−1.1 (3)	N4—C2—C3—C4	0.3 (4)
C7—N3—C9—C10	−179.3 (2)	C1—C2—C3—C4	−179.1 (2)
C11—C10—C9—N3	1.2 (4)	N1—N2—C13—C14	−176.7 (2)
C12—C10—C9—N3	−178.0 (2)	C9—N3—C7—C8	97.7 (3)
C12—N1—N2—C13	−174.3 (2)	C6—N3—C7—C8	−80.4 (3)
C9—C10—C11—O1	179.8 (2)	N2—C13—C14—C19	16.6 (4)
C12—C10—C11—O1	−1.2 (4)	N2—C13—C14—C15	−165.8 (2)
C9—C10—C11—C5	−0.3 (3)	C2—C3—C4—C5	0.0 (4)
C12—C10—C11—C5	178.8 (2)	C6—C5—C4—C3	−0.5 (4)
C4—C5—C11—O1	−0.4 (4)	C11—C5—C4—C3	179.3 (2)
C6—C5—C11—O1	179.4 (2)	N2—N1—C12—O2	4.9 (4)
C4—C5—C11—C10	179.6 (2)	N2—N1—C12—C10	−174.99 (19)
C6—C5—C11—C10	−0.6 (3)	C9—C10—C12—O2	2.0 (4)
C2—N4—C6—N3	179.8 (2)	C11—C10—C12—O2	−177.1 (2)
C2—N4—C6—C5	−0.5 (3)	C9—C10—C12—N1	−178.1 (2)
C9—N3—C6—N4	179.85 (19)	C11—C10—C12—N1	2.8 (3)
C7—N3—C6—N4	−2.0 (3)	C19—C14—C15—C16	−1.0 (4)
C9—N3—C6—C5	0.1 (3)	C13—C14—C15—C16	−178.7 (3)
C7—N3—C6—C5	178.3 (2)	C15—C14—C19—C18	0.9 (5)
C4—C5—C6—N4	0.8 (3)	C13—C14—C19—C18	178.6 (3)
C11—C5—C6—N4	−179.0 (2)	C18—C17—C16—C15	−0.3 (5)
C4—C5—C6—N3	−179.5 (2)	C14—C15—C16—C17	0.7 (5)
C11—C5—C6—N3	0.7 (3)	C16—C17—C18—C19	0.3 (6)
C6—N4—C2—C3	−0.1 (3)	C14—C19—C18—C17	−0.6 (6)
C6—N4—C2—C1	179.3 (2)		

### Hydrogen-bond geometry (Å, °)

**Table:**

*D*—H···*A*	*D*—H	H···*A*	*D*···*A*	*D*—H···*A*
N1—H1N···O1	0.89 (3)	1.93 (2)	2.674 (3)	140 (2)
C7—H7B···O2^i^	0.97	2.45	3.204 (3)	134
C9—H9···O2^i^	0.93	2.51	3.340 (3)	149

Symmetry codes: (i) −*x*+1, −*y*−1, −*z*.
